# Catalytic Diversity of GH30 Xylanases

**DOI:** 10.3390/molecules26154528

**Published:** 2021-07-27

**Authors:** Katarína Šuchová, Vladimír Puchart, Nikolaj Spodsberg, Kristian B. R. Mørkeberg Krogh, Peter Biely

**Affiliations:** 1 Institute of Chemistry, Slovak Academy of Sciences, Dúbravská cesta 9, 84538 Bratislava, Slovakia; Vladimir.Puchart@savba.sk (V.P.); Peter.Biely@savba.sk (P.B.); 2 Novozymes A/S, Krogshøjvej 36, 2880 Bagsværd, Denmark; NSpo@novozymes.com (N.S.); KBK@novozymes.com (K.B.R.M.K.)

**Keywords:** xylanase, glycoside hydrolase family 30, xylan, glucuronoxylanase, xylobiohydrolase

## Abstract

Catalytic properties of GH30 xylanases belonging to subfamilies 7 and 8 were compared on glucuronoxylan, modified glucuronoxylans, arabinoxylan, rhodymenan, and xylotetraose. Most of the tested bacterial GH30-8 enzymes are specific glucuronoxylanases (EC 3.2.1.136) requiring for action the presence of free carboxyl group of MeGlcA side residues. These enzymes were not active on arabinoxylan, rhodymenan and xylotetraose, and conversion of MeGlcA to its methyl ester or its reduction to MeGlc led to a remarkable drop in their specific activity. However, some GH30-8 members are nonspecific xylanases effectively hydrolyzing all tested substrates. In terms of catalytic activities, the GH30-7 subfamily is much more diverse. In addition to specific glucuronoxylanases, the GH30-7 subfamily contains nonspecific endoxylanases and predominantly exo-acting enzymes. The activity of GH30-7 specific glucuronoxylanases also depend on the presence of the MeGlcA carboxyl, but not so strictly as in bacterial enzymes. The modification of the carboxyl group of glucuronoxylan had only weak effect on the action of predominantly exo-acting enzymes, as well as nonspecific xylanases. Rhodymenan and xylotetraose were the best substrates for exo-acting enzymes, while arabinoxylan represented hardly degradable substrate for almost all tested GH30-7 enzymes. The results expand current knowledge on the catalytic properties of this relatively novel group of xylanases.

## 1. Introduction

Glycoside hydrolase (GH) family 30 was found to be a quite diverse family and currently is divided into nine subfamilies [1,2]. Two of them, subfamilies GH30-7 and GH30-8, harbor enzymes that attack β-1,4-d-xylosidic linkages. The subfamily GH30-8 consists mainly of bacterial endo-β-1,4-xylanases specialized for a degradation of xylans containing 4-*O*-methyl-d-glucuronic acid (MeGlcA) or d-glucuronic acid (GlcA) side residues. These so-called glucuronoxylanases cleave the second glycosidic linkage from the uronic-acid-substituted Xyl*p* residue towards the reducing end, generating aldouronic acids of the general formula MeGlcA^2^Xyl_n_ [3,4]. Crystallographic and biochemical data obtained with the enzymes from *Bacillus subtilis* (*Bs*Xyn30C) and *Erwinia chrysanthemi* (*Ec*Xyn30A) provided clear evidence that a conserved arginine in the GH30-8 glucuronoxylanases is crucial to determining their glucuronoxylan specificity, with the guanidine group of the arginine establishing a pair of ionic interactions with the C6 carboxyl group of MeGlcA [5,6]. The importance of the ionic interaction for the action of *Ec*Xyn30A was initially indicated by Hurlbert and Preston [7] and later proven on modified glucuronoxylans, in which the carboxyl groups were either methyl esterified or reduced to 4-*O*-methyl-d-glucose [8,9]. Both modifications caused a several-thousand-fold decrease in catalytic efficiency of *Ec*Xyn30A [8]. In contrast, a substitution of the arginine by alanine caused only 18-fold reduction in the catalytic efficiency which suggested that the substrate specificity of the GH30-8 glucuronoxylanases is determined by an overall topology of the substrate binding site comprising several amino acids [8,10]. 

The availability of the two modified glucuronoxylans deprived of the charged carboxyl group enabled us to perform an analogous study with a larger set of other GH30-8 members but also with a series of recently described eukaryotic GH30-7 enzymes, which show much greater diversity in catalytic properties. In addition to specific glucuronoxylanases, the subfamily GH30-7 includes nonspecific endoxylanases, reducing-end xylose releasing enzymes as well as xylobiohydrolases acting on the nonreducing end. Recent determination of the first 3D structure of the GH30-7 glucuronoxylanase/xylobiohydrolase in complex with a ligand revealed significant differences of how uronic acid moiety is recognized by the eukaryotic xylanases [11]. The specificity determining arginine of the bacterial specific GH30-8 glucuronoxylanases (prokaryotic arginine) is absent in the GH30-7 members and its role is partially substituted by another arginine (eukaryotic arginine) of which only one nitrogen atom of the guanidine group is involved in the ionic interactions with the C6 carboxyl oxygens of MeGlcA. Together with observed differences in β2-α2 loop and topology within the β8-α8 region, the eukaryotic arginine influences the substrate specificity of the GH30-7 enzymes [11,12].

In addition to the comparison of the action of the GH30 enzymes on the modified glucuronoxylans, their activity was also examined on arabinoxylan, rhodymenan, and xylotetraose, i.e., the substrates that do not contain charged uronic acid residues. The results expand current knowledge of the catalytic properties of the GH30 xylanases, and in general, further support the current view on the mode of action and the mechanism of substrate recognition by these unique xylanases.

## 2. Results

### 2.1. Activity of GH30 Xylanases on 4-O-Methylglucuronoxylan and Its Derivatives

**Figure 1:**
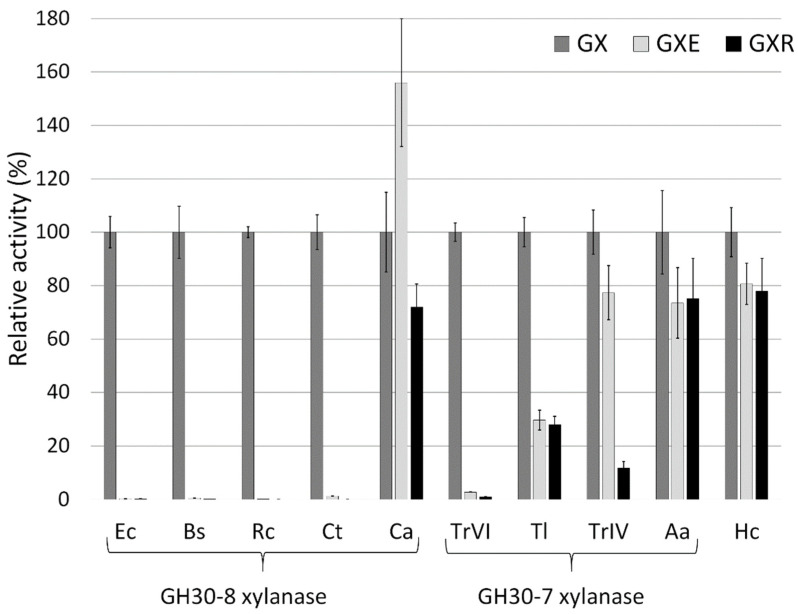
Relative specific activities of GH30-8 and GH30-7 xylanases on GX, GXE and GXR. *Hc*Xyn30A has not yet been classified to any subfamily.

4-*O*-Methylglucuronoxylan (GX) and its two derivatives with eliminated free carboxyl group, 4-*O*-methylglucuronoxylan methyl ester (GXE) and 4-*O*-methylglucoxylan (GXR), were tested as the substrates for several GH30 xylanases (Figure 1). Bacterial GH30-8 xylanases from *Erwinia chrysanthemi Ec*Xyn30A (Ec), *Bacillus subtilis Bs*XynC (Bs), *Ruminococcus champanellensis Rc*Xyn30A (Rc) and *Clostridium themocellum Ct*Xyn30A (Ct) are representatives of glucuronoxylanases (EC 3.2.1.136) requiring for action the presence of free carboxyl group of MeGlcA attached to xylan [3,4,13]. Bacterial enzymes from *Clostridium acetobutylicum Ca*Xyn30A (Ca) and *Hungateiclostridium clariflavum Hc*Xyn30A (Hc) are not specialized for the hydrolysis of GX, but they represent nonspecific xylanase and xylobiohydrolase, respectively [10,14]. Fungal GH30-7 xylanases tested were glucuronoxylanase *Tr*XynVI from *Trichoderma reesei* (TrVI), reducing-end xylose releasing xylanase/endoxylanase *Tr*XynIV from *T. reesei* (TrIV), xylobiohydrolase/endoxylanase *Aa*Xyn30A from *Acremonium alcalophilum* (Aa) and a nonspecific xylanase *Tl*Xyn30A from *Talaromyces leycettanus* (Tl) [15,16,17,18,19]. As expected, specific activity of the glucuronoxylanases *Ec*Xyn30A, *Bs*XynC, *Rc*Xyn30A, *Ct*Xyn30A, and *Tr*XynVI on GXE and GXR were considerably lower than that on GX (Figure 1), confirming that these enzymes require free carboxyl group of the uronic acid residue for their action. On the other hand, activities of *Hc*Xyn30A, *Aa*Xyn30A and *Tr*XynIV on all three substrates were comparable (except of *Tr*XynIV acting on GXR), indicating that the MeGlcA carboxyl group does not play a significant role in the substrate recognition. It is in consonance with a predominant exo-action of these three enzymes [14,16,17]. In the case of *Tl*Xyn30A, the activity on GXE and GXR was approximately three times lower than on GX. This indicates that the free carboxylate may play a certain role in substrate recognition, but it is not indispensable for the enzyme activity. *Ca*Xyn30A was the only xylanase showing a higher activity on GXE than on GX.

**Figure 2:**
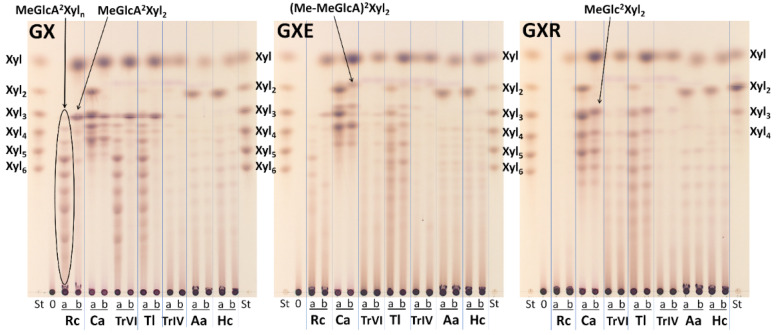
TLC analysis of hydrolysis products of GX, GXE, and GXR by GH30 xylanases. St—standards of linear XOs, a—10 min hydrolysate, b—24 h hydrolysate.

**Figure 3:**
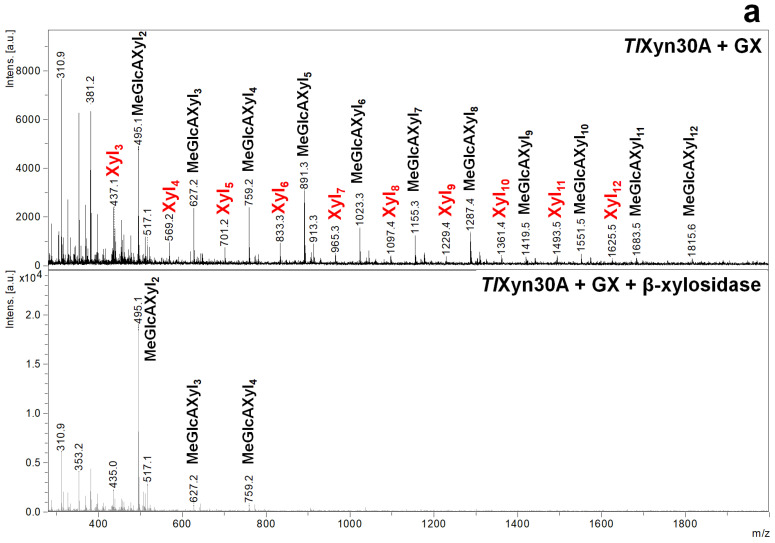

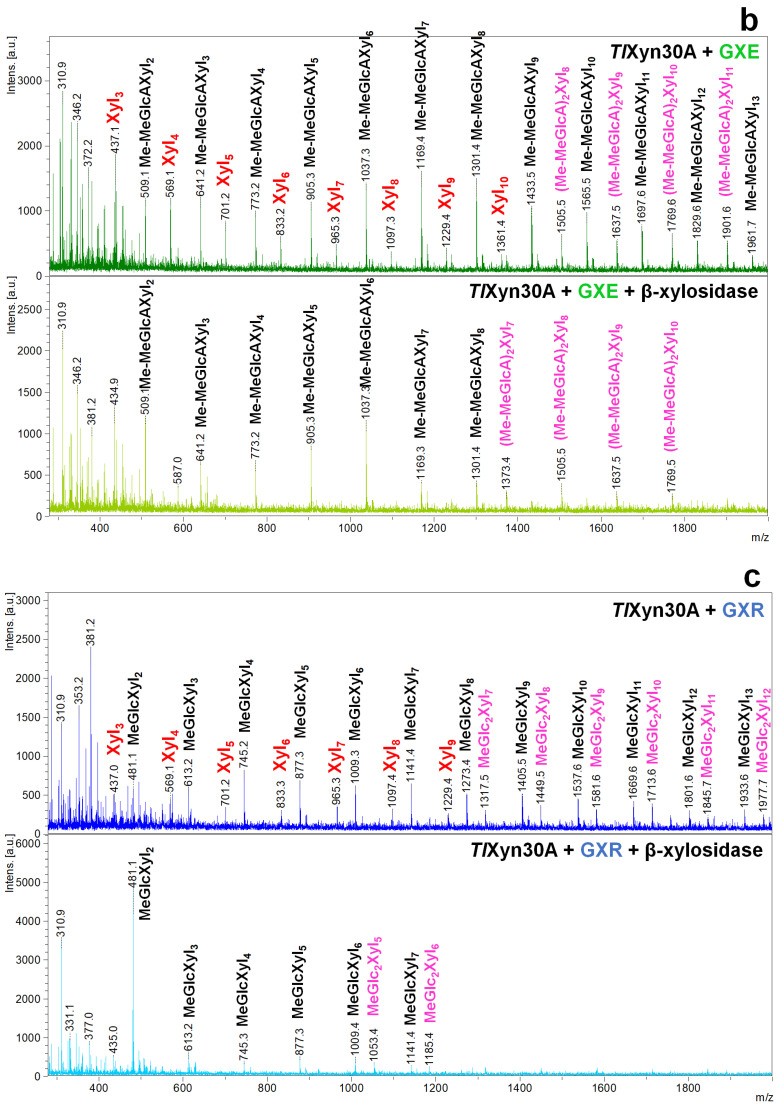
MALDI ToF MS analysis of the hydrolysates of GX (**a**), GXE (**b**) and GXR (**c**) generated by *Tl*Xyn30A and their subsequent hydrolysis by β-xylosidase.

GH30-8 glucuronoxylanases are known to hydrolyze GX to acidic XOs of the general formula MeGlcA^2^Xyl_n_ [3,4]. TLC analysis of hydrolysis products released from GX confirmed the presence of the same products in the hydrolysates generated by *Rc*Xyn30A, *Tr*XynVI, and *Tl*Xyn30A (Figure 2). The products of these three enzymes were shortened to MeGlcA^2^Xyl_2_ upon the hydrolysis with β-xylosidase confirming that the MeGlcA substitution is on the second Xyl*p* residue from the reducing end (Figure 2 and Figure 3a). When GXE or GXR were used as a substrate, *Rc*Xyn30A and *Tr*XynVI liberated only a very low amount of the products (Figure 2). In contrast, GXE and GXR were efficiently cleaved by *Tl*Xyn30A. Interestingly, the products liberated from GXE and GXR by *Tl*Xyn30A were not shortened by β-xylosidase which means that their structure was not analogous to the products released from GX and they do not contain the side residues exclusively on the second Xyl*p* residue from the reducing end but also closer to the nonreducing end (Figure 3b,c). This is in contrast to the action of *Ec*Xyn30A, for which the elimination of the free carboxyl group did not alter the mode of action and consequently the structure of the liberated XOs [8]. The action of *Hc*Xyn30A and *Aa*Xyn30A on GXE and GXR seems to be similar to the action on GX. Xyl_2_ was the main hydrolysis product and the released longer XOs were not attacked by β-xylosidase (Figure 2). *Tr*XynIV generated Xyl, Xyl_2_ and a very small amount of larger XOs from all three substrates.

**Figure 4:**
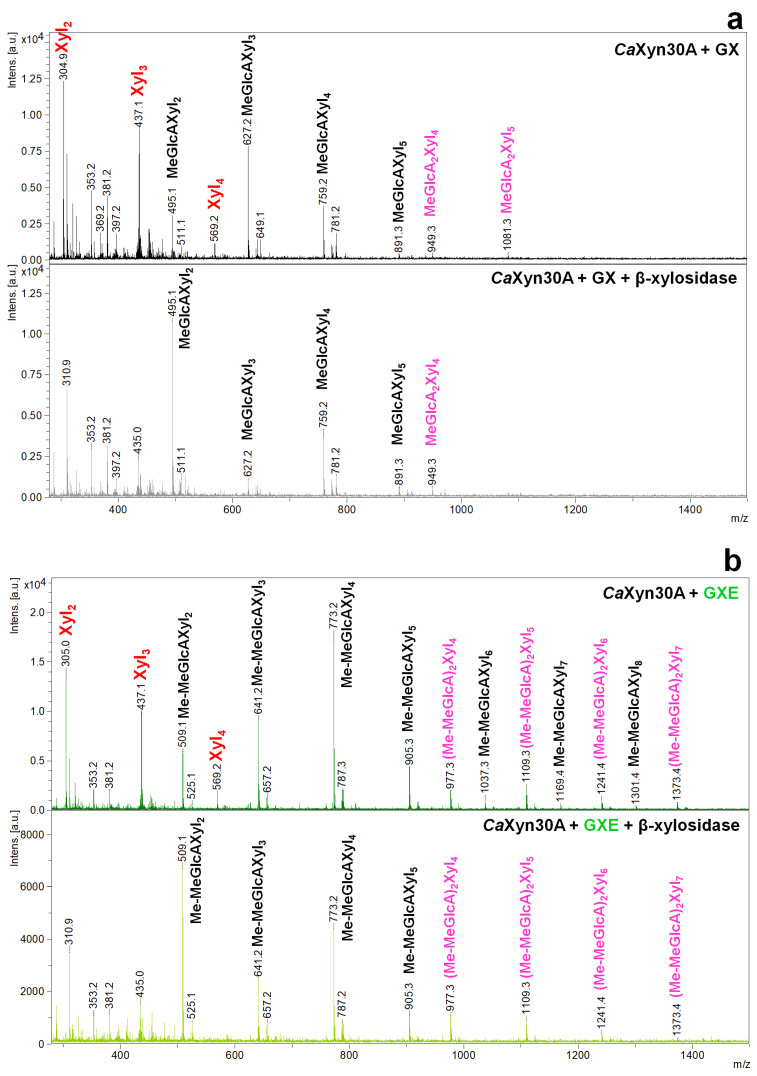

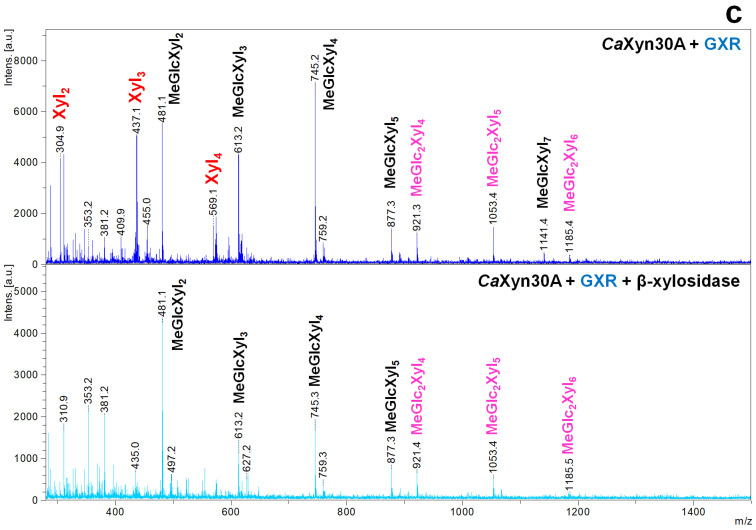
MALDI ToF MS analysis of the hydrolysates of GX (**a**), GXE (**b**) and GXR (**c**) generated by *Ca*Xyn30A and their subsequent hydrolysis by β-xylosidase.

A more detailed analysis was carried out with the products liberated from GX, GXE and GXR by *Ca*Xyn30A. As demonstrated by MALDI-ToF MS analysis, the enzyme generated from all three substrates linear XOs Xyl_2_, Xyl_3_, and Xyl_4_, as well as the branched XOs which were identified as singly substituted Xyl_2_, Xyl_3_, Xyl_4_ and Xyl_5_ (Figure 4a–c). After application of β-xylosidase, linear XOs disappeared, but only some of the branched XOs were shortened to MeGlcA^2^Xyl_2_/Me-MeGlcA^2^Xyl_2_/MeGlc^2^Xyl_2_ and some remained in the hydrolysates. This means that *Ca*Xyn30A liberated products with unsubstituted Xyl*p* unit (s) at the nonreducing end as well as XOs decorated at the nonreducing end.

### 2.2. Action of GH30 Xylanases on Rho, AraX and Xyl_4_

**Figure 5:**
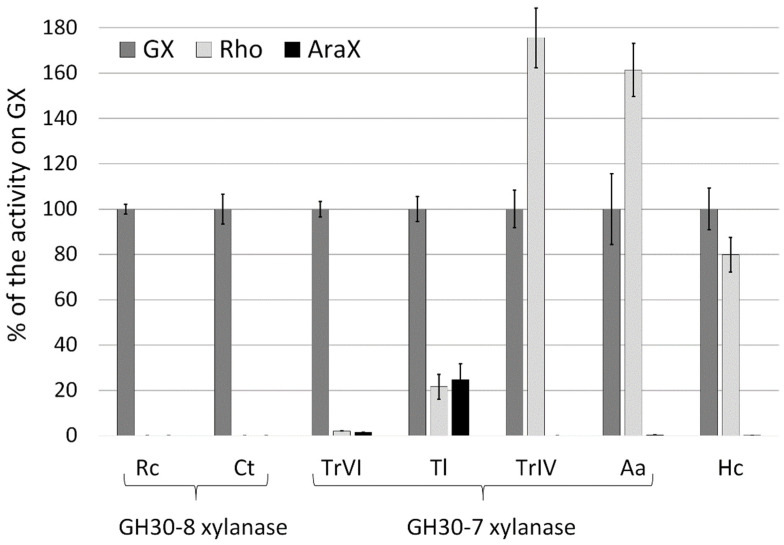
Relative specific activities of selected GH30 xylanases on Rho and AraX. The activity on GX is taken as 100%.

The specific activities of the GH30 xylanases on GX were compared with those on natural uncharged polysaccharides—linear β-1,3-β-1,4-xylan (rhodymenan, Rho) and wheat arabinoxylan (AraX) (Figure 5, Table 1). Glucuronoxylanases *Rc*Xyn30A and *Ct*Xyn30A did not hydrolyze Rho and AraX, and very low levels of activity were observed with *Tr*XynVI. *Hc*Xyn30A exhibited similar activity on GX and Rho, while the activity of *Tr*XynIV and *Aa*Xyn30A on Rho was about 1.6-fold higher than on GX. *Tl*Xyn30A exhibited on Rho and AraX about 20–25% of the activity on GX, and it was the only examined xylanase showing a significant activity on AraX. For other tested xylanases AraX represents hardly degradable substrate (Figure 5). *Ca*Xyn30A (not tested on AraX in this study) was reported to cleave AraX efficiently [10].

**Figure 6:**
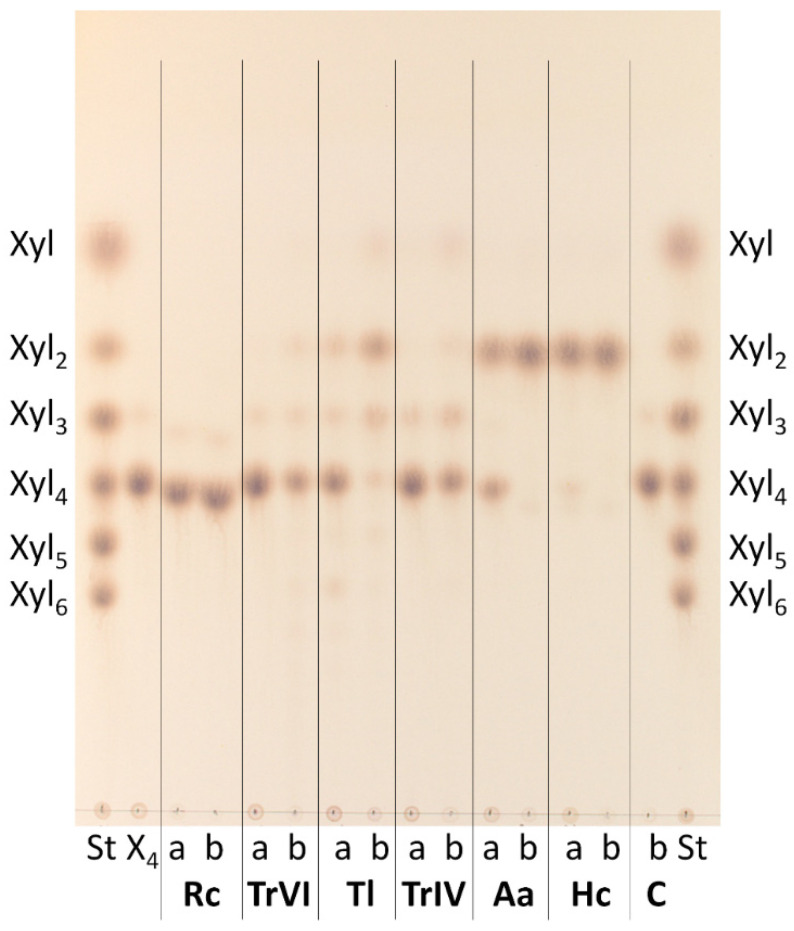
TLC analysis of hydrolysis products of Xyl_4_ generated by GH30 xylanases. St—standards of linear XOs, a—15 min hydrolysate, b—24 h hydrolysate, C—Xyl_4_ control without any enzyme incubated for 24 h.

Xyl_4_ was not hydrolyzed by *Rc*Xyn30A, and it was only very slowly attacked by *Tr*XynVI where the hydrolysis was accompanied by a generation of transglycosylation products (Figure 6). On the other hand, the tetrasaccharide was rapidly and exclusively cleaved to xylobiose by *Aa*Xyn30A and *Hc*Xyn30A. *Tr*XynIV slowly released xylose. *Tl*Xyn30A generated Xyl_2_ as the main product with a small amount of Xyl and Xyl_3_. During this conversion, a production of XOs having higher degree of polymerization than the substrate was observed, indicating transglycosylation reactions. All tested enzymes released xylose from the reducing end of MeGlcA^3^Xyl_4_ except of *Aa*Xyn30A and *Hc*Xyn30A which did not attack this substrate (data not shown).

### 2.3. Effect of MeGlcA Content on an Extent of GX Hydrolysis

**Figure 7:**
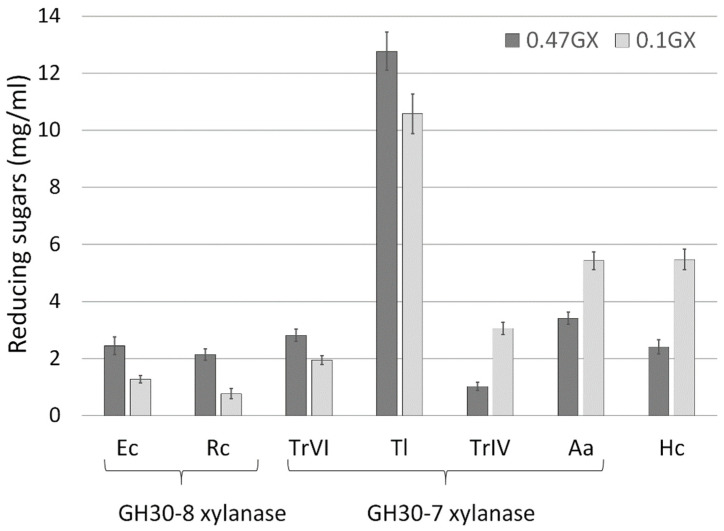
Final level of reducing sugars released from beechwood GXs containing 0.47 µmol MeGlcA/mg (0.47GX) or 0.1 µmol MeGlcA/mg (0.1GX) by GH30 xylanases. The substrates were incubated with a high load of the enzymes for 7 days.

The action of GH30 xylanases was compared on beechwood GX with different content of MeGlcA—0.47 µmol MeGlcA/mg and 0.1 µmol MeGlcA/mg (Figure 7). The intention was to find out how the degree of MeGlcA substitution affects the final amount of the reducing sugars. As expected, the amount of reducing sugars released by *Ec*Xyn30A, *Rc*Xyn30A and *Tr*XynVI was higher from the more substituted GX, since the action of these enzymes is strictly dependent on MeGlcA content. On the other hand, predominantly exo-acting enzymes released more reducing sugars from the less substituted GX, despite its poorer solubility in comparison with the more substituted counterpart. Compared to the other enzymes, *Tl*Xyn30A released the highest amount of reducing sugars from both GXs (Figure 7).

## 3. Discussion

The catalytic properties of the tested xylanases are summarized in Table 1. The performance of the GH30-7 and GH30-8 members on GX, GXE and GXR confirmed the necessity of the free carboxyl group attached to the substrate main chain for the effective action of GH30-8 glucuronoxylanases—*Ec*Xyn30A, *Bs*XynC, *Ct*Xyn30A and *Rc*Xyn30A, as well as GH30-7 glucuronoxylanase *Tr*XynVI. All these enzymes released products of the general formula MeGlcA^2^Xyl_n_ which were shortened to MeGlcA^2^Xyl_2_ upon incubation with β-xylosidase, as previously shown for other glucuronoxylanases [3,4,13,20]. The enzymes acting predominantly by exo-fashion (*Hc*Xyn30A, *Aa*Xyn30A, *Tr*XynIV) were not influenced by the modification of the carboxyl group of MeGlcA and their action was stopped at the first substitution regardless of its nature. This is in consonance with the fact that the branched products released from all three substrates by *Hc*Xyn30A and *Aa*Xyn30A were not shortened by β-xylosidase indicating that the side residues were located at or close to the non-reducing end of the products. About three times higher activity of *Tl*Xyn30A on GX than on GXE and GXR suggests that the enzyme somehow recognizes the carboxyl group of the substrates, but its esterification or reduction does not end the enzyme activity. *Tl*Xyn30A contains an Arg residue corresponding to the Arg46 of *Tc*Xyn30B which was shown to interact ionically with the carboxyl group of the substrate [11], and which may contribute to the recognition of MeGlcA substitution by *Tl*Xyn30A. Interestingly, the products released by *Tl*Xyn30A from GXE and GXR differed from those released from GX. They did not contain the side chain exclusively on the second Xyl*p* residue from the reducing end. *Tl*Xyn30A recognizes MeGlcA substitution in the –2b subsite, but if the charged substituent is absent, the enzyme allows an accommodation of the substituted Xyl*p* residue also in other than the −2 subsite.

A comparison of the hydrolysis rates of MeGlcA^3^Xyl_4_ and Xyl_4_ by *Ca*Xyn30A showed that Xyl_4_ was hydrolyzed faster than its substituted analog [10]. On the other hand, an analogous comparison of the enzyme action on linear and Ara-substituted XOs revealed that 2-*O*-arabinosylated compounds are markedly better substrates than the corresponding linear XOs [10]. This indicates that *Ca*Xyn30A does not recognize MeGlcA substitution of the Xyl*p* unit in the −2 subsite but α-1,2-linked Ara*f* on the xylose in the −2 subsite contributes to a tighter binding of arabinosylated XOs. The activity of *Ca*Xyn30A on GX, GXE and GXR confirms that the type of decoration at Xyl*p* residue accommodated in the –2 subsite affects the enzyme activity and acidic substituent may not be favorable in this subsite. However, the partial resistance of the products released from GX, GXE and GXR to the action of β-xylosidase suggests, that the substituents of the main chain of the substrates may be accommodated in various subsites.

The activity of GH30 xylanases on Rho, AraX and Xyl_4_ further confirmed that polymeric and oligomeric substrates lacking MeGlcA decoration are poor substrates for glucuronoxylanases *Rc*Xyn30A and *Tr*XynVI. Very low or no activity on AraX was reported for some other glucuronoxylanases (Table 1 and Table 2). Rho was better substrate than GX for *Aa*Xyn30A and *Tr*XynIV and equally good for *Hc*Xyn30A. Xylobiohydrolases *Aa*Xyn30A and *Hc*Xyn30A were shown to cleave also β-1,3-linkages which may contribute to better hydrolysis of Rho [14,17]. The lower extent of Rho and AraX hydrolysis by *Tl*Xyn30A in comparison to GX also supports the hypothesis that MeGlcA substitution is somehow recognized by *Tl*Xyn30A, but its presence is not crucial for the enzyme activity.

From all tested enzymes, only *Tl*Xyn30A efficiently hydrolyzed AraX. The ability to cleave AraX was reported for several GH30 enzymes (Table 1 and Table 2). Four enzymes (*Ca*Xyn30A, *Cp*Xyn30A from *Ruminiclostridium papyrosolvens*, *Tc*Xyn30C and *Tc*Xyn30A from *Talaromyces cellulolyticus*), all lacking the prokaryotic or eukaryotic Arg, exhibited even higher specific activities on AraX than on GX [10,24,29,30]. However, the specific activities of these enzymes varied a lot (from 0.279 to 113 U/mg), and only AraX hydrolysis by *Tc*Xyn30C, and particularly *Ca*Xyn30A can be designated as effective. Specific activities of *Talaromyces (Penicillium) purpurogenus Tp*Xyn30A on GX and AraX were comparable, while activity of XYLD from *Bispora* sp. on AraX was about 30% of the activity on GX [27,28]. These two enzymes seem to be nonspecific xylanases not recognizing any substitution of the xylan main chain.

Glucuronoxylanases generally do not cleave linear XOs, and only a very high enzyme loadings lead to a weak hydrolysis [9,21,22]. On the contrary, linear XOs were good substrates for xylobiohydrolases *Hc*Xyn30A and *Aa*Xyn30A, glucuronoxylanases/xylobiohydrolases *Tc*Xyn30B, *Tt*Xyn30A from *Thermothelomyces thermophila*, as well as for nonspecific GH30-7 xylanases *Tl*Xyn30A, *Tc*Xyn30C, *Tp*Xyn30A, and GH30-8 xylanases *Ca*Xyn30A and *Cp*Xyn30A (Figure 5, Table 1 and Table 2). Reducing-end xylose-releasing exoxylanases *Tr*XynIV and *Tc*Xyn30A also efficiently cleaved linear XOs. The specific activity of *Tc*Xyn30A on Xyl_3_ was two orders of magnitude higher than on polymeric substrates indicating that the short linear XOs are preferred substrates for this xylanase [29]. On the other hand, nonspecific GH30 xylanases preferred longer XOs (Xyl_5_ and Xyl_6_) over shorter ones (Table 2).

The experiment in which the extent of hydrolysis was compared on two GXs with different MeGlcA content showed that *Tl*Xyn30A released the highest amount of reducing sugars. In contrast to glucuronoxylanases or exo-xylanases, *Tl*Xyn30A is able to hydrolyze substituted as well as unsubstituted parts of xylan chain, which makes it an interesting candidate in the processes where the high extent of hydrolysis is required. More information about the versatile catalytic capability of *Tl*Xyn30A can be found in the accompanying paper [19].

As a summary of this study we can say that GH30-8 members are mostly specific glucuronoxylanases showing poor activity on the substrates without MeGlcA side residues. The conversion of MeGlcA to its methyl ester or its reduction to MeGlc, leads to a remarkable drop in the specific activity of these enzymes. Exceptions are *Ca*Xyn30A and *Cp*Xyn30A, which do not contain prokaryotic Arg and of which the substrate binding sites differ from that of bacterial glucuronoxylanases. The catalytic properties of GH30-7 members are more diverse. The mode of action and activity of GH30-7 glucuronoxylanases is also determined by the interaction of the MeGlcA carboxyl group with another Arg; however, this interaction does not appear to be so strong as that in GH30-8 subfamily. The modifications of the carboxyl group do not influence the action of predominantly exo-acting enzymes, as well as nonspecific xylanases. However, it may change the cleavage mode of the modified polysaccharides. The catalytic properties of individual enzymes must therefore be appraised before their specific application.

## 4. Materials and Methods

### 4.1. Substrates, Standards and Enzymes

4-*O*-Methylglucuronoxylan (GX), 4-*O*-methylglucuronoxylan methyl ester (GXE), 4-*O*-methylglucoxylan (GXR) and aldopentaouronic acid MeGlcA^3^Xyl_4_ were prepared as described earlier [8,31,32]. To guarantee the same branching pattern in all three xylan derivatives, the GXR and GX were prepared from the same batch of soluble GXE fraction by a reduction and alkaline deesterification, respectively. Rhodymenan, an algal linear β-1,3-β-1,4-xylan from *Palmaria palmata*, was a gift from the laboratory of Prof. M. Claeyssens (University of Ghent, Ghent, Belgium). Wheat arabinoxylan (Ara:Xyl 38:62) and linear β-1,4-xylooligosaccharides (Xyl_2_–Xyl_6_) were purchased from Megazyme International (Bray, Ireland). Xylose was from Serva (Heidelberg, Germany). GX with 0.1% content of MeGlcA was from Lenzing (Lenzing, Austria). β-Xylosidase was a recombinant *Aspergillus niger* enzyme from GH3 family expressed in *Saccharomyces cerevisiae* [33]. Enzymes *Ec*Xyn30A (42 kDa), *Bs*XynC (44 kDa), *Tr*XynIV (43 kDa), *Tr*XynVI (57 kDa), *Tl*Xyn30A (55 kDa), *Aa*Xyn30A (58 kDa) were prepared as described previously [5,15,16,17,18,34]. *Ct*Xyn30A (54 kDa) (product number: CZ0445) was purchased from NZYTech, *Hc*Xyn30A (58 kDa) (product number: CZ0916) and *Rc*Xyn30A (48 kDa) (product number: CZ10281) were a generous gift of Prof. Carlos M.G.A. Fontes (NZYTech, Lisboa, Portugal) and *Ca*Xyn30A (39 kDa) was generously donated by Dr. F. J. St John (Institute for Microbial and Biochemical Technology, Forest Products Laboratory, USDA Forest Service, Madison, WI, USA).

### 4.2. Hydrolysis of Polysaccharides and Oligosaccharides

Polysaccharides (GX, GXE, GXR, Rho, AraX) were used in a concentration of 10 mg.mL^−1^ in 0.05 M sodium phosphate buffer, pH 7, for *Rc*Xyn30A, *Ct*Xyn30A, *Bs*Xyn30A, *Aa*Xyn30A, *Hc*Xyn30A, and in 0.05 M sodium acetate buffer, pH 4, for *Ca*Xyn30A, *Tr*XynVI, *Tr*XynIV, *Tl*Xyn30A. Enzymes were appropriately diluted (25 nM–4.4 µM) and 1 µL was mixed with 20 µL of polysaccharide solution and incubated at 37 °C for 20 or 60 min. Beechwood GXs (300 µL) containing 0.47 µmol MeGlcA/mg and 0.1 µmol MeGlcA/mg were incubated at 37 °C for 7 days under a layer of toluene. The reducing sugars were determined by the Somogyi–Nelson procedure [35]. All reactions were done in triplicate. One unit of enzyme activity is defined as the amount of the enzyme releasing 1 µmol of reducing sugars expressed as an equivalent of xylose in 1 min. For TLC analysis, 5 µL of the mixtures were spotted on silica gel coated aluminum sheets (Merck, Darmstadt, Germany) after 10 min and 24 h of hydrolysis. After 24 h, the reaction was terminated by 5 min heating at 100 °C, followed by an overnight treatment with β-xylosidase (1 U mL^−1^) at 37 °C. pH was adjusted to 4.0 with 4 M acetic acid in the phosphate buffered samples prior β-xylosidase addition (due to a lower pH optimum of the β-xylosidase). 10 mM solution of Xyl_4_ (10 µL) in appropriate buffer (see above) was mixed with 10 µL of 4.8 µM enzymes and 1.5 µL was spotted on silica gel coated aluminum sheets after 15 min and 24 h of hydrolysis at 37 °C. TLC plates were developed twice in the solvent system ethyl acetate/acetic acid/2-propanol/formic acid/water 25:10:5:1:15 (*v*/*v*) and the sugars were visualized with orcinol reagent (0.5% orcinol in 5% sulphuric acid in ethanol).

Protein concentration was determined by the Bradford method [36].

### 4.3. MALDI ToF MS

The hydrolysates of GX, GXE and GXR were decationized by Dowex 50 (H^+^ form) and 1 µL was mixed with 1 µL of the matrix (1% solution of 2,5-dihydroxybenzoic acid in 30% acetonitrile) directly on MS target plate. After air-drying, the samples were analyzed by UltrafleXtreme MALDI ToF/ToF mass spectrometer (Bruker Daltonics, Bremen, Germany) operating in reflectron positive mode.

## Figures and Tables

**Table 1:** Performance of GH30 xylanases on Rho, AraX and GX and its uncharged modifications GXE and GXR. Abbreviations: P, presence of prokaryotic Arg; E, presence of eukaryotic Arg and N, absence of both Args.

	GH30 Subfamily	Arg	Specific Activity (U/mg)			% of Activity on GX * Exhibited on		Products Released From			Reference
			GX	Rho	AraX	GXE	GXR	GX	GXE	GXR	
*Ec*Xyn30A	8	P	46.7	nd	nd	0.3	0.3	MeGlcA^2^Xyl_n_	Me-MeGlcA^2^Xyl_n_	MeGlc^2^Xyl_n_	[8]
*Bs*XynC	8	P	59.5 ^a^	nd	nd	0.6	0.2	MeGlcA^2^Xyl_n_	Me-MeGlcA^2^Xyl_n_	MeGlc^2^Xyl_n_	^a^ [3], this study
*Ct*Xyn30A	8	P	17 ^b^	nd	nd	1.4	nd	MeGlcA^2^Xyl_n_	Me-MeGlcA^2^Xyl_n_	MeGlc^2^Xyl_n_	^b^ [12], this study
*Rc*Xyn30A	8	P	21.7	nd	nd	0.03	nd	MeGlcA^2^Xyl_n_	Me-MeGlcA^2^Xyl_n_	MeGlc^2^Xyl_n_	this study
*Ca*Xyn30A	8	N	90.9 ^c^	nt	113 ^c^	156	72	Xyl_2_-Xyl_4_, MeGlcA(Xyl)_2–5_	Xyl_2_-Xyl_4_, Me-MeGlcA(Xyl)_2__–__5_	Xyl_2_-Xyl_4_, MeGlc(Xyl)_2__–5_	^c^ [13], this study
*Tr*XynVI	7	E	5.2	0.1	0.078	2.8	1.1	Xyl_2_-Xyl_4_, MeGlcA^2^Xyl_n_	Xyl_2_-Xyl_4_, Me-MeGlcA^2^Xyl_n_	Xyl_2_-Xyl_4_, MeGlc^2^Xyl_n_	this study
*Tl*Xyn30A	7	E	12.4	3.1	3.5	29.7	28	Xyl_n_, MeGlcA^2^Xyl_n_	Xyl_n_, Me-MeGlcA(Xyl)_n_	Xyl_n_, MeGlc(Xyl)_n_	this study
*Tr*XynIV	7	N	0.11	0.18	nd	77.4	11.8	Xyl, Xyl_2_, MeGlcA(Xyl)_n_	Xyl, Xyl_2_, Me-MeGlcA(Xyl)_n_	Xyl, Xyl_2_, MeGlc(Xyl)_n_	this study
*Aa*Xyn30A	7	E	3.2 ^d^	5.4 ^d^	0.09 ^d^	73.5	75.2	Xyl_2_, MeGlcA^n^^−1^Xyl_n_, MeGlcA^n^Xyl_n_	Xyl_2_, Me-MeGlcA^n^^−^^1^Xyl_n_, Me-MeGlcA^n^Xyl_n_	Xyl_2_, MeGlc^n^^−^^1^Xyl_n_, MeGlc^n^Xyl_n_	^d^ [17], this study
*Hc*Xyn30A	?	N	13.4 ^e^	10.7 ^e^	0.011 ^e^	80.6	78.1	Xyl_2_, MeGlcA^n^^−1^Xyl_n_, MeGlcA^n^Xyl_n_	Xyl_2_, Me-MeGlcA^n^^−^^1^Xyl_n_, Me-MeGlcA^n^Xyl_n_	Xyl_2_, MeGlc^n^^−^^1^Xyl_n_, MeGlc^n^Xyl_n_	^e^ [14], this study

nd—not detected, nt—not tested, * activity on GX was taken as 100%, ^a–e^ data from the references given in the last column.

**Table 2:** Specific activities of characterized GH30 xylanases on beech GX, wheat AraX and action of the enzymes on linear XOs. Abbreviations: P, presence of prokaryotic Arg; E, presence of eukaryotic Arg and N, absence of both Args.

	GH30 Subfamily	Arg	Specific Activity (U/mg)		Products and Rates of Linear XOs Hydrolysis	Specificity	Reference
			Beech GX	AraX			
*Bl*Xyn30A	8	P	7.87	nd	products Xyl, Xyl_2_, Xyl_3_, very low rate	glucuronoxylanase	[21]
*Bs*LC9Xyn30	8	P	36.2	nt	products Xyl_2_, Xyl_3_, very low rate	glucuronoxylanase	[22]
*Pb*Xyn30A	8	P	30.3	nd	nt	glucuronoxylanase	[20]
*Pf*Xyn30A	8	P	244	nd	nt	glucuronoxylanase	[23]
*Cp*Xyn30A	8	N	1.1 *	1.7	products Xyl_2_, Xyl_3_ and Xyl_4_, Xyl_6_-1.19 U/mg, Xyl_5_-0.36 U/mg, Xyl_4_-very low rate	nonspecific xylanase	[24]
*Tt*Xyn30A	7	E	6	0.07	products Xyl_2_ (even XOs) or Xyl and Xyl_2_ (odd XOs)	glucuronoxylanase/xylobiohydrolase	[25]
*Tc*Xyn30B	7	E	11.3	nd	products Xyl_2_ (even XOs) or Xyl and Xyl_2_ (odd XOs), Xyl_3_-0.388 U/mg	glucuronoxylanase/xylobiohydrolase	[26]
*Tp*Xyn30A	7	E	24	22	product mainly Xyl_2_	presumably nonspecific xylanase	[27]
*Bis*XYLD	7	E	2463	790	nt	nonspecific xylanase	[28]
*Tc*Xyn30A	7	N	0.162	0.279	product Xyl, Xyl_3_-28.1 U/mg	reducing-end xylose releasing exoxylanase	[29]
*Tc*Xyn30C	7	N	38	47	products mainly Xyl_2_ and Xyl_3_, Xyl_6_-1.6 U/mg, Xyl_5_-0.42 U/mg, Xyl_4_-0.131 U/mg, Xyl_3_-0.015 U/mg	nonspecific xylanase	[30]

* Value for sweetgum glucuronoxylan, nt—not tested, nd—not detected.

## Data Availability

Not applicable.

## Sample Availability

Samples of the compounds are not available from the authors.
